# Mineral encapsulation of microorganisms in calcified oral biofilms: implications for immune dysregulation and vascular calcification

**DOI:** 10.3389/fdmed.2026.1849108

**Published:** 2026-06-03

**Authors:** Jayalaxmi Shetty, Nishmitha N. Hegde, Mithra N. Hegde

**Affiliations:** Nitte (Deemed to be University), AB Shetty Memorial Institute of Dental Sciences (ABSMIDS), Department of Conservative Dentistry and Endodontics, Mangalore, India

**Keywords:** calcium phosphate, cardiovascular disease, hydroxyapatite crystals, inflammation, microbial persistence, mineral encapsulation, oral health, oral microbiome

## Abstract

Oral biofilms represent highly organized microbial ecosystems embedded within extracellular matrices enriched with calcium and phosphate ions that promote the nucleation of calcium phosphate minerals, including hydroxyapatite. During plaque mineralization, microorganisms may become incorporated within calcium phosphate–protein matrices, forming mineralized microenvironments that facilitate microbial persistence while partially shielding pathogens from host immune surveillance. **Hydroxyapatite crystals can also directly influence innate immune responses**. Macrophages exposed to these particles exhibit altered polarization, impaired antigen presentation, and sustained low-grade inflammatory signaling accompanied by dysregulated tissue repair mechanisms. In biological fluids, calcium phosphate nanoparticles rapidly acquire a protein corona that modulates cellular uptake, biodistribution, and systemic interactions. These particles may disrupt intracellular calcium homeostasis, promote endothelial dysfunction, influence coagulation pathways, and contribute to vascular remodeling. We propose that calcium phosphate mineralization within oral biofilms encapsulates microbial cells within mineral–protein matrices that behave as protected reservoirs capable of systemic dissemination, immune modulation, and promotion of vascular calcification. This mineral encapsulation model provides a mechanistic framework linking opportunistic oral microorganisms with chronic inflammation and cardiovascular disease and suggests potential targets for therapeutic intervention.

## 1 Introduction

The oral cavity represents a dynamic interface between host tissues and environmental microorganisms and harbors one of the most complex microbial ecosystems in the human body. Certain anatomical structures within the oral environment, particularly teeth and alveolar bone, lack conventional lymphatic drainage, which may influence immune surveillance and microbial clearance. As a result, microorganisms inhabiting oral biofilms can employ immune-evasion strategies such as molecular mimicry and immune modulation, enabling their long-term persistence within host tissues.

Increasing evidence indicates that oral pathogens may disseminate systemically and contribute to vascular disease. Several studies have detected oral bacterial DNA in vascular lesions, suggesting a relationship between oral infections and cardiovascular disorders such as atherosclerosis and abdominal aortic aneurysms (1, 2). Periodontal pathogens including *Porphyromonas gingivalis, Prevotella intermedia*, and *Tannerella forsythia* have been identified in atherosclerotic plaques and vascular tissues (3–7). Similarly, the cariogenic bacterium *Streptococcus mutans* has been detected in heart valve tissues and atheromatous plaques, indicating a potential role of opportunistic microbes in cardiovascular pathology (3, 8, 9). Oral bacteria may enter the bloodstream through inflamed periodontal tissues and disseminate hematogenously to distant organs, including the vascular system. This dissemination is supported by the detection of bacterial DNA and microbial components within cardiovascular tissues (10, 11). Periodontal infections can also induce systemic inflammatory responses characterized by endothelial dysfunction, immune activation, and pro-thrombotic states, which accelerate atherosclerotic plaque development (6, 11, 12).

Vascular calcification is increasingly recognized as an actively regulated biological process involving osteogenic differentiation of vascular smooth muscle cells and deposition of calcium phosphate minerals within the vascular wall. This process contributes to arterial stiffness, plaque instability, and progression of cardiovascular disease (13).

**Figure 1:**
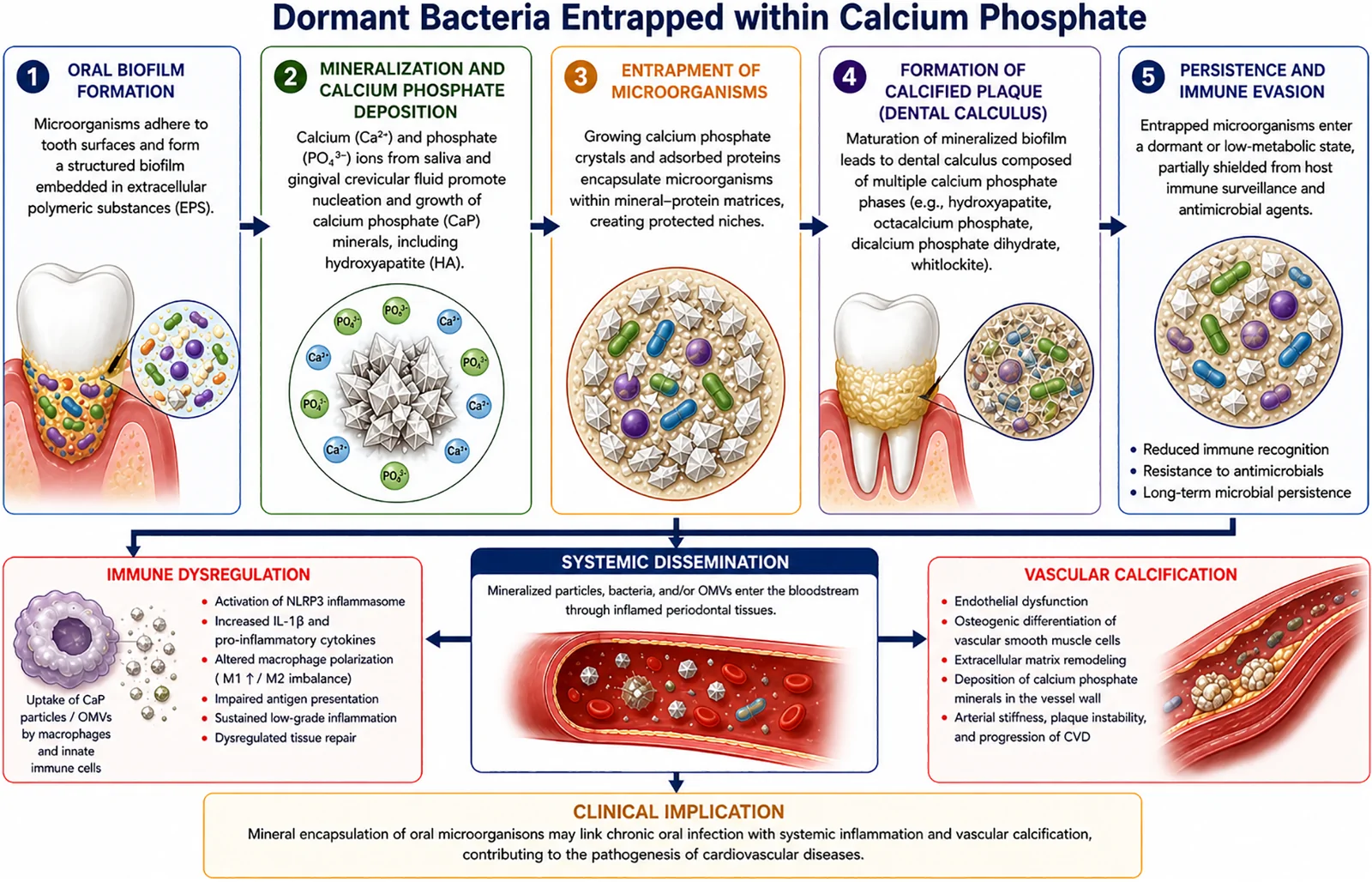
Flow chart for pathogen encapsulation in calcified plaque and its role in immune dysregulation and vascular calcification.

Despite growing evidence linking oral infections with cardiovascular disease, the mechanistic pathways by which mineralized oral biofilms contribute to vascular calcification remain poorly understood. We propose that mineralization within oral biofilms leads to calcium phosphate–mediated encapsulation of microbial pathogens, creating mineral–protein matrices that facilitate microbial persistence, immune modulation, systemic dissemination, and promotion of vascular calcification. The proposed mechanism linking calcified oral biofilms, microbial persistence, immune dysregulation, and vascular calcification is summerised in Figure 1.

## 2 Dental plaque and calcium phosphate mineralization

The oral microbiota consists of highly diverse microbial communities dominated by bacterial phyla such as Firmicutes, Bacteroidetes, Proteobacteria, Actinobacteria, and Fusobacteria. Common oral bacterial genera include Streptococcus, Actinomyces, Veillonella, Fusobacterium, Porphyromonas, and Prevotella. In addition to bacteria, the oral ecosystem also contains fungi such as Candida species, archaea, protozoa, and viruses including bacteriophages, all of which contribute to biofilm ecology and host–microbe interactions (14–21).

**Dental plaque is a structured microbial biofilm composed of microorganisms embedded** within extracellular polymeric substances enriched in calcium and phosphate ions. These ions promote nucleation and growth of calcium phosphate minerals, including hydroxyapatite. As plaque undergoes mineralization, it forms dental calculus, a hardened structure containing multiple calcium phosphate phases such as hydroxyapatite, octacalcium phosphate, dicalcium phosphate dihydrate, and whitlockite (22–24). Amorphous calcium phosphate acts as a precursor that gradually transforms into hydroxyapatite, the most thermodynamically stable crystalline phase (22, 25). During mineralization, microorganisms can become embedded within mineral–protein matrices that stabilize the biofilm architecture and protect bacteria from environmental stresses and antimicrobial agents (22, 26, 27). Certain bacteria, such as *Corynebacterium matruchotii*, contribute to intracellular calcification processes that promote the aggregation of calcified material and microbial components within calculus (28).

**Calcium phosphate (CaP) particles generated during mineralization exhibit high surface reactivity and can interact with biomolecules such as proteins, nucleic acids, and lipids through electrostatic interactions and molecular binding** (29–31). Their biological behavior is influenced by physicochemical characteristics including particle size, morphology, crystallinity, and surface charge (32, 33). These particles can be internalized by host cells through endocytic pathways including clathrin-mediated endocytosis, caveolae-mediated uptake, and macropinocytosis. Smaller particles exhibit enhanced cellular uptake and may evade lysosomal degradation, allowing interaction with intracellular signaling pathways (34–36).

## 3 Immune recognition of calcium phosphate crystals

Calcium phosphate crystals, including basic calcium phosphate (BCP) and calcium pyrophosphate dihydrate (CPPD), are increasingly recognized as biologically active structures capable of triggering innate immune responses. A central mechanism involves activation of the NLRP3 inflammasome, which leads to secretion of pro-inflammatory cytokines such as interleukin-1β (IL-1β) (37–40). These mediators amplify inflammatory signaling and contribute to tissue injury.

Macrophages play a critical role in responding to these crystals. These cells exhibit considerable functional plasticity and can transition between resting (M0), pro-inflammatory (M1), and anti-inflammatory (M2) phenotypes depending on environmental signals (41, 42). Upon encountering calcium phosphate crystals, macrophages, along with monocytes and neutrophils, internalize or interact with the particles, triggering oxidative stress and cellular injury pathways that may culminate in apoptosis or necrosis (38, 39). Crystal recognition is mediated in part by pattern recognition receptors such as macrophage receptor with collagenous structure (MARCO) and low-density lipoprotein receptor (LDLR), which facilitate particle binding and uptake (43). Proteins interact with CaP surfaces through ionic bonds, hydrogen bonds, and electrostatic attractions. For instance, acidic amino groups on proteins bind to Ca^2^⁺ ions, while alkaline amino groups interact with phosphate groups (PO₄^3^⁻) (44, 45). When calcium phosphate particles interact with biological fluids, they rapidly acquire a protein corona composed of adsorbed plasma proteins. This corona influences particle stability, cellular uptake, and immunological responses (46). Surface characteristics including crystal phase composition, microporosity, and surface area strongly influence protein adsorption (47, 48). Crystal deposition also promotes reactive oxygen species (ROS) generation, which further amplifies inflammatory signaling and contributes to tissue damage (49).

## 4 Oral microbial species associated with vascular calcification

Several oral microorganisms have been implicated in vascular inflammation and calcification. *Porphyromonas gingivalis*, a key periodontal pathogen, has been detected in atherosclerotic plaques and can induce endothelial dysfunction and osteogenic signaling in vascular smooth muscle cells (50, 51).

*Fusobacterium nucleatum*, an important mediator of biofilm maturation, enhances polymicrobial invasion and inflammatory responses associated with vascular pathology (52, 53). *Eikenella corrodens* has also been identified in carotid atherosclerotic plaques and is associated with symptomatic vascular disease (52).

Oral streptococci such as *Streptococcus oralis* and *Streptococcus sanguinis* have also been linked to cardiovascular conditions, including infective endocarditis and aortic valve disease (53). Additionally, *Aggregatibacter actinomycetemcomitans* contributes to vascular pathology through platelet activation and thrombus formation (54, 55). Calcifying nanoparticles, sometimes referred to as nanobacteria, have also been proposed as potential initiators of pathological mineralization processes in both oral and vascular tissues (56, 57).

Bacterial outer membrane vesicles (OMVs) provide another mechanism connecting oral infection with systemic disease. These nanoscale vesicles carry virulence factors such as lipopolysaccharide and enzymes that can stimulate inflammatory signaling, modulate host gene expression, and promote platelet aggregation (58).

## 5 Emerging concept: mineral-associated microbial persistence

**Building upon these observations, an emerging concept suggests that mineralization** processes occurring within oral biofilms generate calcium phosphate structures capable of entrapping microbial cells or microbial components. These mineralized microenvironments may function as protected reservoirs that allow microbial persistence while partially shielding pathogens from immune surveillance. When mineral particles enter systemic circulation, they interact with plasma proteins to form mineral–protein complexes that influence biodistribution and cellular uptake.

Experimental studies demonstrate that microporous hydroxyapatite surfaces adsorb significantly higher amounts of proteins such as fibronectin and serum albumin compared with non-microporous materials (59). This enhanced adsorption alters cell attachment, morphology, and downstream cellular responses (59–61). Calcium phosphate nanoparticles isolated from dental plaque have been shown to contain proteins such as elongation factor-Tu and fetuin-A, both of which promote mineral nucleation and stabilization (62). Following cellular uptake, these particles may undergo lysosomal degradation, resulting in elevated intracellular calcium levels (55–57). These findings are further supported by studies investigating intracellular delivery and cellular trafficking of calcium phosphate nano particle (88, 89).

Disruption of intracellular calcium homeostasis can trigger inflammatory signaling, induce cellular stress or apoptosis, and promote osteogenic differentiation of vascular smooth muscle cells, ultimately contributing to vascular calcification. Although experimental evidence remains limited, mineral-associated microbial persistence may represent a previously underrecognized mechanism linking oral microbial biofilms with chronic vascular inflammation and calcification.

## 6 Clinical implications

Recognition of the relationship between oral microbial ecosystems and vascular health has important implications for disease prevention and therapy.

Maintaining oral hygiene and effective periodontal care can reduce systemic inflammatory burden and improve vascular outcomes. Clinical studies demonstrate that treatment of periodontal disease improves endothelial function and reduces inflammatory markers, emphasizing the importance of oral health in cardiovascular disease prevention (63–65).

Strategies to reduce plaque mineralization may also limit the formation of calcified biofilms. Inhibitors such as phytate and zinc incorporated into oral care products can reduce calcium phosphate crystallization and dental calculus formation (66). Microbiome-modulating approaches are emerging as additional therapeutic strategies. Probiotics, particularly species of *Lactobacillus* and *Bifidobacterium*, may help restore microbial balance, reduce inflammation, and improve periodontal health (67–69).

Dietary patterns further influence oral microbial communities. Diets rich in plant-based foods, fiber, and bioactive compounds—such as the Mediterranean diet—are associated with reduced pathogenic microbial species and lower systemic inflammation, whereas high-sugar diets promote microbial dysbiosis (68, 70–73).

## 7 Future directions

Future research should focus on clarifying the molecular mechanisms linking oral microbial ecosystems with vascular mineralization and identifying translational strategies for early detection and intervention.

Integration of multi-omics technologies, including genomics, epigenomics, metabolomics, and transcriptomics, will be essential for identifying regulatory pathways involved in vascular calcification (74, 75). Noncoding RNAs such as microRNAs, long noncoding RNAs, and circular RNAs are emerging as important regulators of calcification pathways and represent promising therapeutic targets (76, 77).

Advances in imaging technologies are improving detection of early calcification events. Molecular imaging probes capable of binding hydroxyapatite can enable visualization of initial mineral deposition (78, 79), while positron emission tomography using ^18F-NaF allows detection of microcalcifications associated with high-risk plaques (80–82). **Complementary approaches, including automated immunoassays detecting calcified OMVs and tissue-nonspecific alkaline phosphatase activity, may provide minimally invasive biomarkers for early calcification** (83). Further mechanistic studies should investigate calcification-regulating pathways such as pyrophosphate metabolism and alkaline phosphatase activity (84). Modulation of inflammatory signaling and development of nanotechnology-based drug delivery systems may provide targeted therapeutic strategies for preventing vascular calcification (85). Emerging gene-editing technologies, including CRISPR-based approaches guided by multi-omics data, may enable precise targeting of genetic and epigenetic drivers of vascular calcification (86). Computational and systems-biology approaches integrating imaging, molecular, and multi-omics datasets may provide a comprehensive framework for understanding calcification processes and predicting disease progression (87).

## 8 Conclusion

Mineralization within oral biofilms represents a complex biological process with potential systemic consequences. The mineral encapsulation model proposed here suggests that calcium phosphate deposition within oral microbial communities may entrap pathogens within mineral–protein matrices that function as protected reservoirs. These mineralized structures may facilitate microbial persistence, promote immune dysregulation, and contribute to vascular calcification through inflammatory signaling and disruption of cellular calcium homeostasis.

Understanding these interactions may provide new insights into the connection between oral health and cardiovascular disease and identify novel therapeutic targets for preventing pathological vascular calcification.
